# Age and Seasonal Variation in Allergen Sensitivity Among Children: A Comprehensive Analysis of Food and Environmental Allergens

**DOI:** 10.7759/cureus.104947

**Published:** 2026-03-09

**Authors:** Bilal Hashmi, Asma Sherazi, Lena Jafri, Ali Saleem, Nasreen Bano, Hafsa Majid

**Affiliations:** 1 Pathology and Laboratory Medicine, Aga Khan University, Karachi, PAK; 2 Pediatrics and Child Health, Aga Khan University, Karachi, PAK

**Keywords:** allergen-specific-ige, allergy, environmental, environmental allergy, food, pediatric

## Abstract

Background

Allergic conditions, particularly food and environmental allergies, are increasingly recognized as important contributors to pediatric morbidity worldwide. However, data describing age-related and seasonal patterns of allergen sensitization in children from low- and middle-income countries remain limited. This retrospective, cross-sectional, laboratory-based study aimed to evaluate the frequency and seasonal variation of food and environmental allergen sensitization among children undergoing allergen-specific IgE testing.

Methods

A total of 2,796 pediatric patients (1,676 males and 1,120 females) under 18 years of age who underwent allergen-specific IgE testing between January 2013 and July 2023 were included. Participants were categorized into four age groups: <1, 2-5, 6-10, and 11-18 years. Six food allergens and eight environmental allergens were analyzed using the IMMULITE^®^ 2000 system (Siemens Healthcare Diagnostics Inc., Deerfield, IL, USA) with 3gAllergy^®^ kits. Specific IgE ≥0.35 kU/L was considered positive. Associations between allergen positivity, age, sex, and season were assessed using chi-square and Kruskal-Wallis tests.

Results

*Dermatophagoides farinae* (52.5%) and *Dermatophagoides pteronyssinus* (52.1%) were the most frequently detected environmental allergens, while egg white (30%) and shrimp (28.7%) were the most common food allergens. Environmental allergen positivity was higher in younger children, whereas food allergen positivity peaked in adolescents (11-18 years). Male participants demonstrated higher overall allergen positivity rates. Significant seasonal variation was observed, with increased sensitization during autumn and winter months.

Conclusions

This study characterizes patterns of allergen sensitization within a clinically tested pediatric cohort in Pakistan, demonstrating age- and season-related variation in allergen-specific IgE positivity. As a laboratory-based analysis, the findings reflect immunologic sensitization rather than confirmed clinical allergy and should not be interpreted as population-level prevalence. These data provide region-specific insights that may support clinical evaluation and patient counseling in similar settings.

## Introduction

Allergic diseases affect approximately 22% of the global population and pose a significant healthcare burden [1]. Global epidemiological data reveal a substantial rise in the prevalence of allergic disorders over the past few decades [2]. The term “allergy” was first introduced in 1906 by Viennese pediatrician Clemens von Pirquet, who observed that some of his patients exhibited hypersensitivity to substances such as dust, pollen, and certain foods [3]. Allergen-specific IgE plays a key role in the development of allergic disorders, as an allergic reaction occurs after an individual becomes sensitized to an allergen through IgE. Identifying allergens before sensitization is crucial for diagnosing allergic diseases in patients with a history of allergies [4]. Effective treatment and prevention depend on accurately identifying the causative allergens and implementing appropriate avoidance measures [5].

The ongoing rise in air pollution and climate change, driven by urbanization and increased energy consumption, increases human exposure to a variety of pollutants and allergens. Children frequently encounter allergens, including pollen, pet dander, and specific foods, during critical stages of development. The distribution of allergens varies according to environmental factors such as climate, seasonal changes, and lifestyle elements, including dietary habits and the presence of pets in different geographic regions [6]. Furthermore, sensitivity to specific allergens can differ across population subsets, including variations by age and sex [7]. The global prevalence of food allergies in children is estimated at approximately 4% and varies based on age, geographic location, and individual dietary habits [8,9]. Food allergies are triggered by immune reactions in individuals sensitive to particular foods. One study conducted in Pakistan reported elevated reactivity to key environmental and food allergens in patients with high total IgE levels [10].

Pakistan is a developing country facing a substantial burden of endemic and epidemic infectious diseases, as well as noncommunicable diseases. In this context, the primary focus is on infectious diseases, while allergic conditions are often overlooked. Currently, there is no national registry to track the prevalence of allergic diseases associated with environmental and dietary factors. Misdiagnosed or untreated childhood allergies can lead to significant health-related problems in adulthood, such as asthma, which has a prevalence of 4.3% in Pakistan [11-13]. This study aimed to analyze the prevalence of common environmental and food allergens and their age- and season-related variation among children in Pakistan. Recognizing these patterns can provide evidence-based support for strategies to prevent and manage allergic diseases.

This article was previously presented as a meeting abstract at EUROMEDLAB 2025 on May 18-22, 2025.

## Materials and methods

This retrospective cross-sectional laboratory-based study was conducted in the Section of Chemical Pathology, Department of Pathology and Laboratory Medicine, in collaboration with the Department of Pediatrics and Child Health at Aga Khan University, Karachi, Pakistan. Ethical approval was obtained from the Ethics Review Committee of Aga Khan University (approval 2024-9840-28481). The study was conducted in accordance with the Declaration of Helsinki, and all patient identifiers were removed to ensure confidentiality.

Laboratory data for allergen-specific IgE (sIgE) testing in pediatric patients (<18 years) from January 2013 to July 2023 were extracted from the Laboratory Information Management System. All pediatric patients who underwent allergen-specific IgE testing during the study period were included. Aga Khan University’s laboratory network consists of a central reference laboratory in Karachi, supported by stat laboratories in eight additional cities and approximately 300 phlebotomy centers across nearly 100 cities nationwide. Samples were processed centrally using standardized operating procedures.

Allergen-specific IgE testing was performed on serum samples using chemiluminescence immunoassay technology on the IMMULITE^®^ 2000 system (Siemens Healthcare Diagnostics Inc., Deerfield, IL, USA) with 3gAllergy^®^ kits. The analytical measurement range for sIgE was 0.10-100 kU/L. A specific IgE level ≥0.35 kU/L was considered positive, in accordance with the manufacturer’s recommendations.

Six food allergens (peanut, milk, shrimp, soybean, egg white, and beef) and eight environmental allergens (cat dander, dog epithelium, cockroach, *Dermatophagoides pteronyssinus*, *Dermatophagoides farinae*, *Penicillium notatum*, *Aspergillus fumigatus*, and *Russian thistle*) were analyzed. Specific IgE reactivity was categorized into manufacturer-defined classes. For analytical purposes, Class I and above (≥0.35 kU/L) were considered positive. Internal quality control procedures included both normal and abnormal controls with each analytical batch. Throughout the study period, the laboratory participated in external proficiency testing programs conducted by the College of American Pathologists, ensuring analytical accuracy and inter-laboratory standardization.

Statistical analysis

Descriptive and inferential statistical methods were applied using Stata version 14 (StataCorp LLC, College Station, TX, USA). Duplicate samples were removed from the dataset. Age was categorized into four groups (<1 year, 2-5 years, 6-10 years, and 11-18 years), and seasonal variations were divided into four groups (spring, summer, autumn, and winter). Frequency tables were constructed for categorical data, and descriptive statistics were calculated for continuous data. Chi-square tests were used to evaluate associations between allergen reactivity and variables such as age, sex, and seasonality.

## Results

A total of 2,796 participants (1,676 males and 1,120 females) were tested for allergen-specific IgE levels during the study period. Participants were divided into four age groups: <1 year, 2-5 years, 6-10 years, and 11-18 years. The majority of tests were conducted during the autumn (N = 804, 28.7%) and winter (N = 760, 27.2%) seasons, as summarized in Table 1.

**Table 1:** Comparison of positive allergen-specific IgE reactivities by gender, season, and age group

Allergen type	Female, N (%)	Male, N (%)	(χ²)	p-Value	Season				(χ²)	p-Value (χ²)	Age (years)				(χ²)	p-Value (χ²)
					Spring, N (%)	Summer, N (%)	Autumn, N (%)	Winter, N (%)			<1, N (%)	2-5, N (%)	6-10, N (%)	11-18, N (%)		
Total study subjects	1120 (40.1)	1676 (59.9)	4.057	0.044	682 (24.4)	550 (19.7)	804 (28.7)	760 (27.2)	3.69	0.297	559 (20)	995 (35.6)	649 (23.2)	593 (21.2)	≥16.266	<0.001
Environmental allergens																
Cat dander	486 (42.9)	648 (57.1)	6.0	0.013	266 (23.5)	242 (21.3)	325 (28.7)	301 (26.5)	3.7	0.30	330 (59.0)	380 (38.2)	230 (35.4)	194 (32.7)	103.6	<0.001
Cockroach	473 (42.8)	632 (57.2)	5.6	0.017	267 (24.2)	232 (21.0)	306 (27.7)	300 (27.1)	2.4	0.50	327 (58.5)	378 (38.0)	227 (35.0)	173 (29.2)	117.4	<0.001
Dermatophagoides farinae	597 (40.6)	873 (59.4)	0.4	0.53	344 (23.4)	312 (21.2)	429 (29.2)	385 (26.2)	6.4	0.09	329 (58.9)	479 (48.1)	359 (55.3)	303 (51.1)	19.2	<0.001
Dog epithelium	466 (43.1)	615 (56.9)	6.6	0.009	260 (24.1)	230 (21.3)	303 (28.0)	288 (26.6)	2.9	0.41	329 (58.9)	378 (38.0)	211 (32.5)	163 (27.5)	137.9	<0.001
Aspergillus fumigatus	471 (43.2)	619 (56.8)	7.2	0.007	267 (24.5)	230 (21.1)	303 (27.8)	290 (26.6)	2.7	0.45	329 (58.9)	379 (38.1)	216 (33.3)	166 (28.0)	132.1	<0.001
Penicillium notatum	472 (43.4)	615 (56.6)	8.2	0.004	261 (24.0)	229 (21.1)	305 (28.1)	292 (26.9)	2.2	0.53	329 (58.9)	380 (38.2)	211 (32.5)	167 (28.2)	133.8	<0.001
Russian thistle	473 (42.6)	637 (57.4)	4.8	0.025	261 (23.5)	236 (21.3)	314 (28.3)	299 (26.9)	3.1	0.37	329 (58.9)	385 (38.7)	217 (33.4)	179 (30.2)	119.2	<0.001
Dermatophagoides pteronyssinus	590 (40.4)	869 (59.6)	0.2	0.67	339 (23.2)	313 (21.5)	425 (29.1)	382 (26.2)	7.9	0.04	329 (58.9)	472 (47.4)	353 (54.4)	305 (51.4)	20.4	<0.001
Food allergens																
Egg white	341 (40.5)	500 (59.5)	0.1	0.73	197 (23.4)	167 (19.9)	244 (29.0)	233 (27.7)	0.6	0.89	114 (20.4)	252 (25.3)	182 (28.0)	293 (49.4)	142.3	<0.001
Soybean	314 (40.4)	463 (59.6)	0.0	0.81	181 (23.3)	142 (18.3)	231 (29.7)	223 (28.7)	2.9	0.41	82 (14.7)	219 (22.0)	184 (28.4)	292 (49.2)	200.6	<0.001
Beef	315 (40.6)	460 (59.4)	0.1	0.69	187 (24.1)	145 (18.7)	229 (29.5)	214 (27.6)	0.8	0.84	86 (15.4)	218 (21.9)	182 (28.0)	289 (48.7)	190.0	<0.001
Milk	307 (40.6)	450 (59.4)	0.1	0.74	188 (24.8)	141 (18.6)	226 (29.9)	202 (26.7)	1.2	0.76	72 (12.9)	214 (21.5)	182 (28.0)	289 (48.7)	213.9	<0.001
Peanut	317 (39.9)	478 (60.1)	0.0	0.9	184 (23.1)	153 (19.2)	233 (29.3)	225 (28.3)	1.4	0.70	85 (15.2)	229 (23.0)	186 (28.7)	295 (49.7)	194.8	<0.001
Shrimp	322 (40.1)	481 (59.9)	0.0	0.98	192 (23.9)	145 (18.1)	240 (29.9)	226 (28.1)	2.5	0.48	79 (14.1)	228 (22.9)	195 (30.0)	301 (50.8)	215.7	<0.001

**Figure 1:**
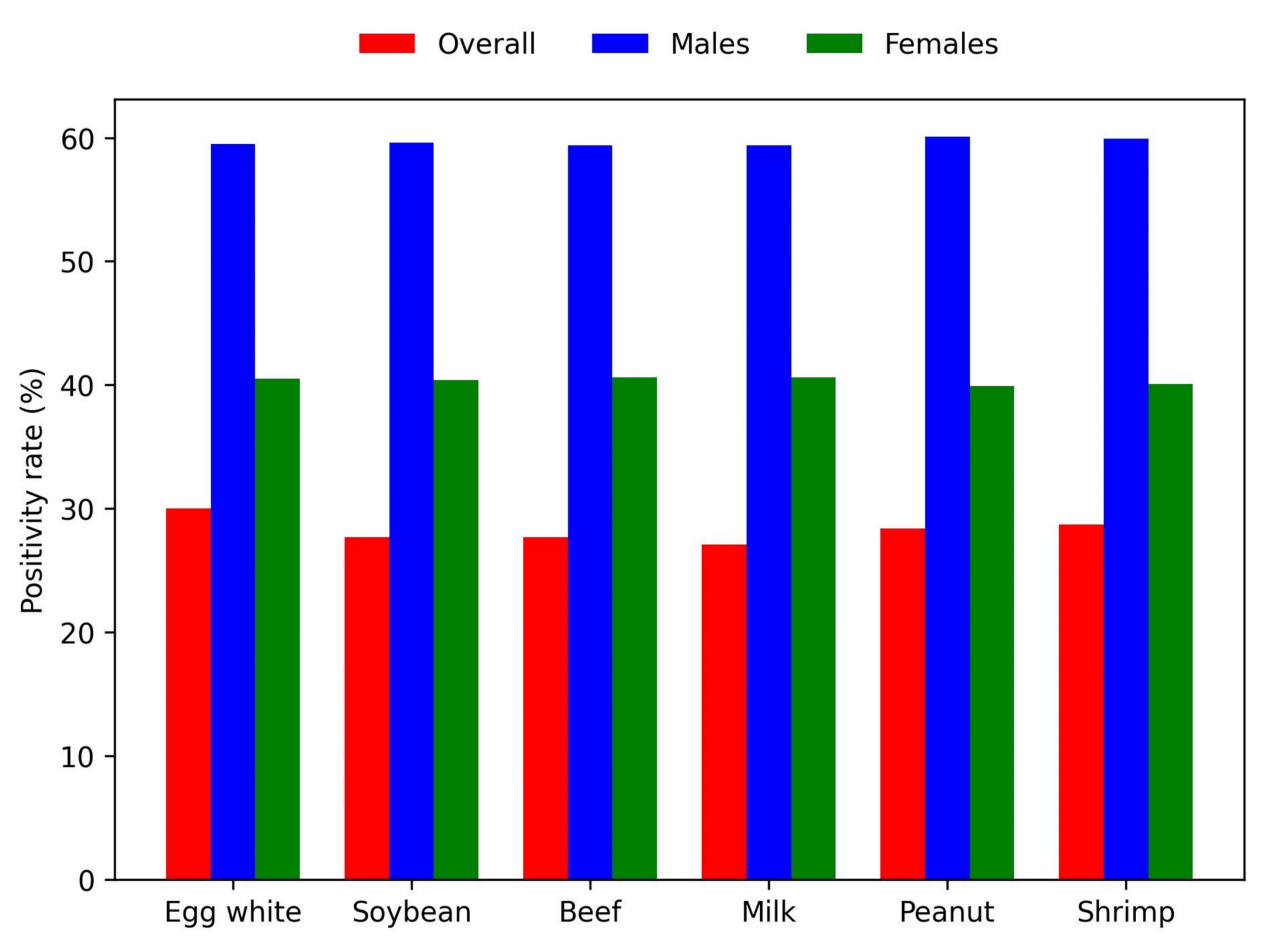
Food allergen positivity rates

**Figure 2:**
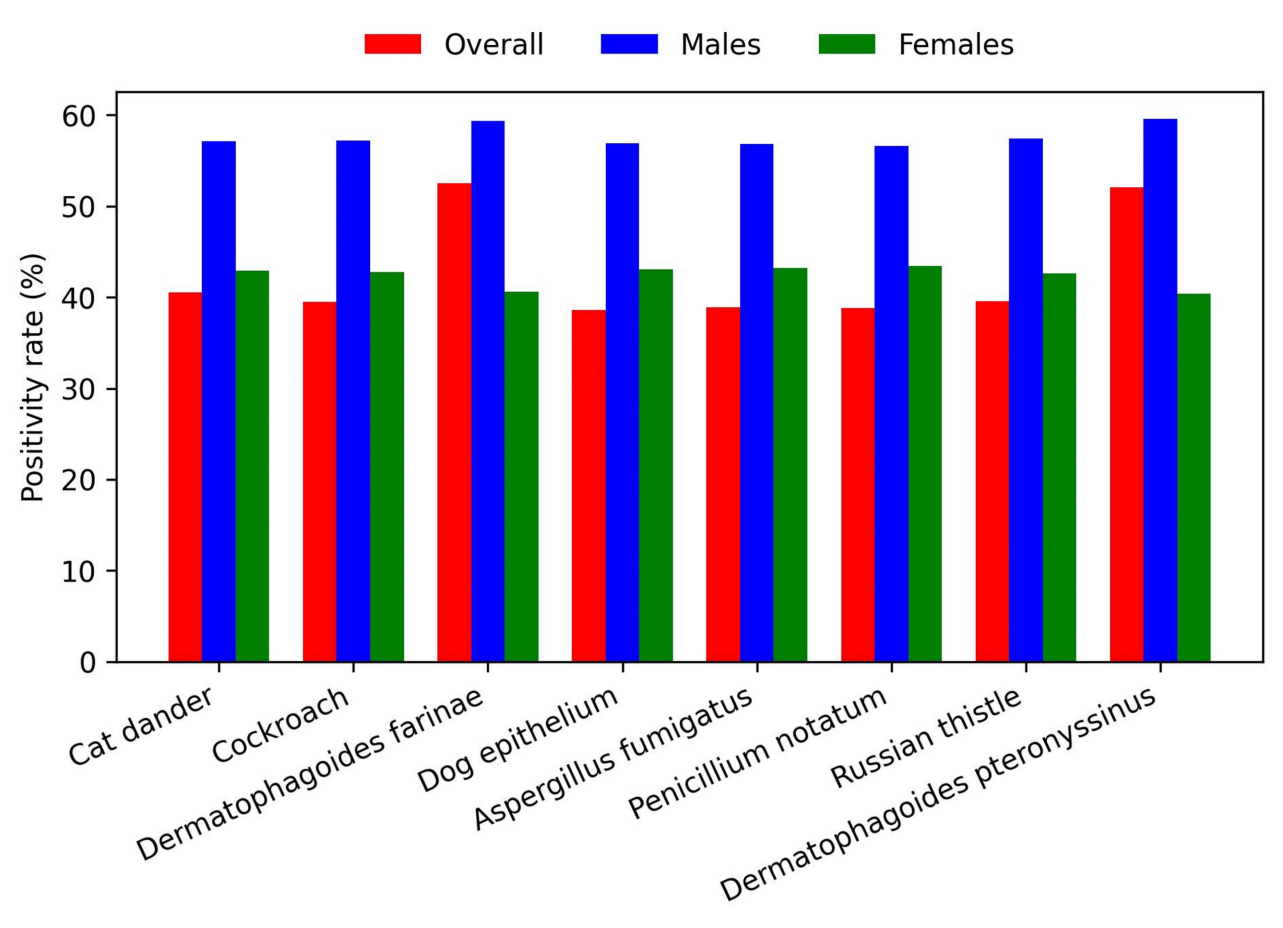
Environmental allergen positivity rates

**Figure 3:**
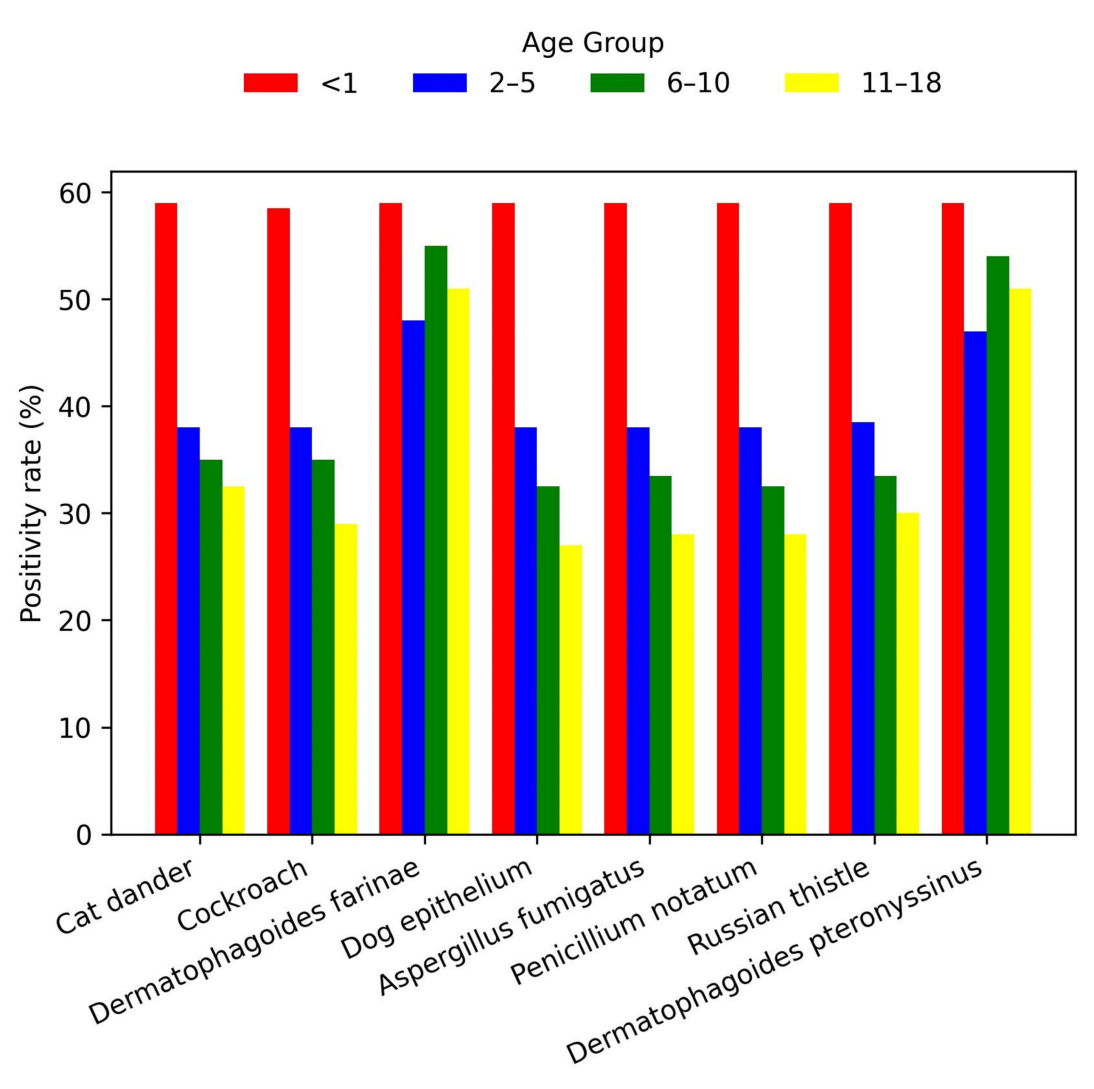
Environmental allergens by age group

**Figure 4:**
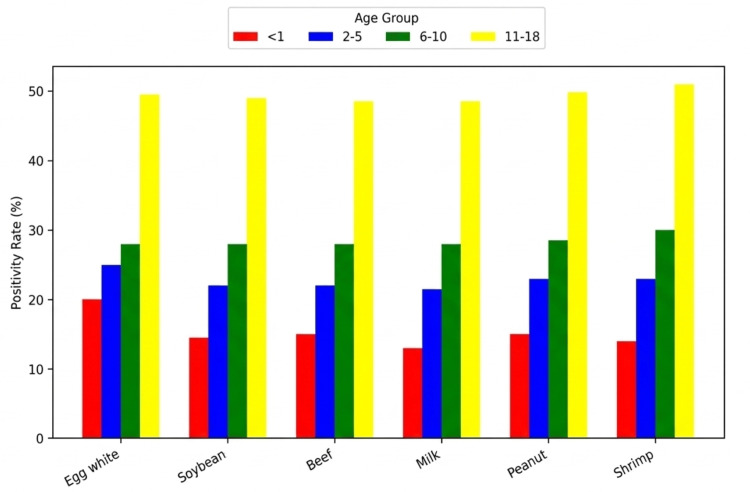
Food allergens by age group

**Figure 5:**
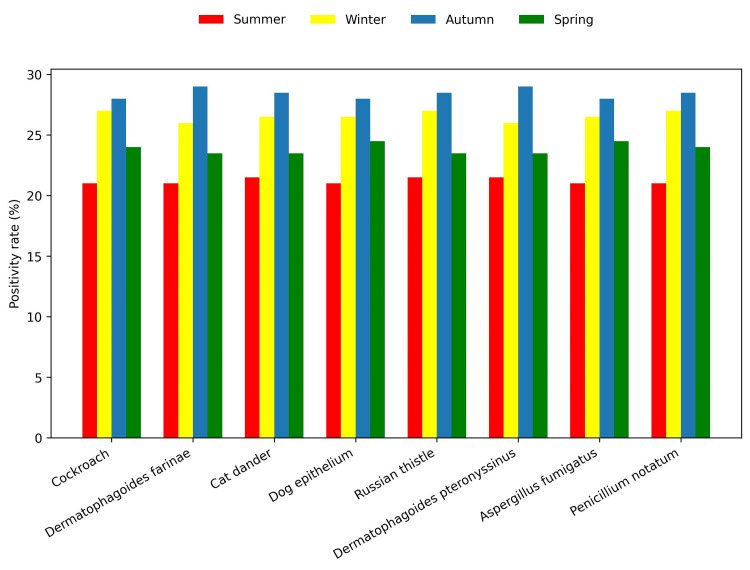
Environmental allergens by season

**Figure 6:**
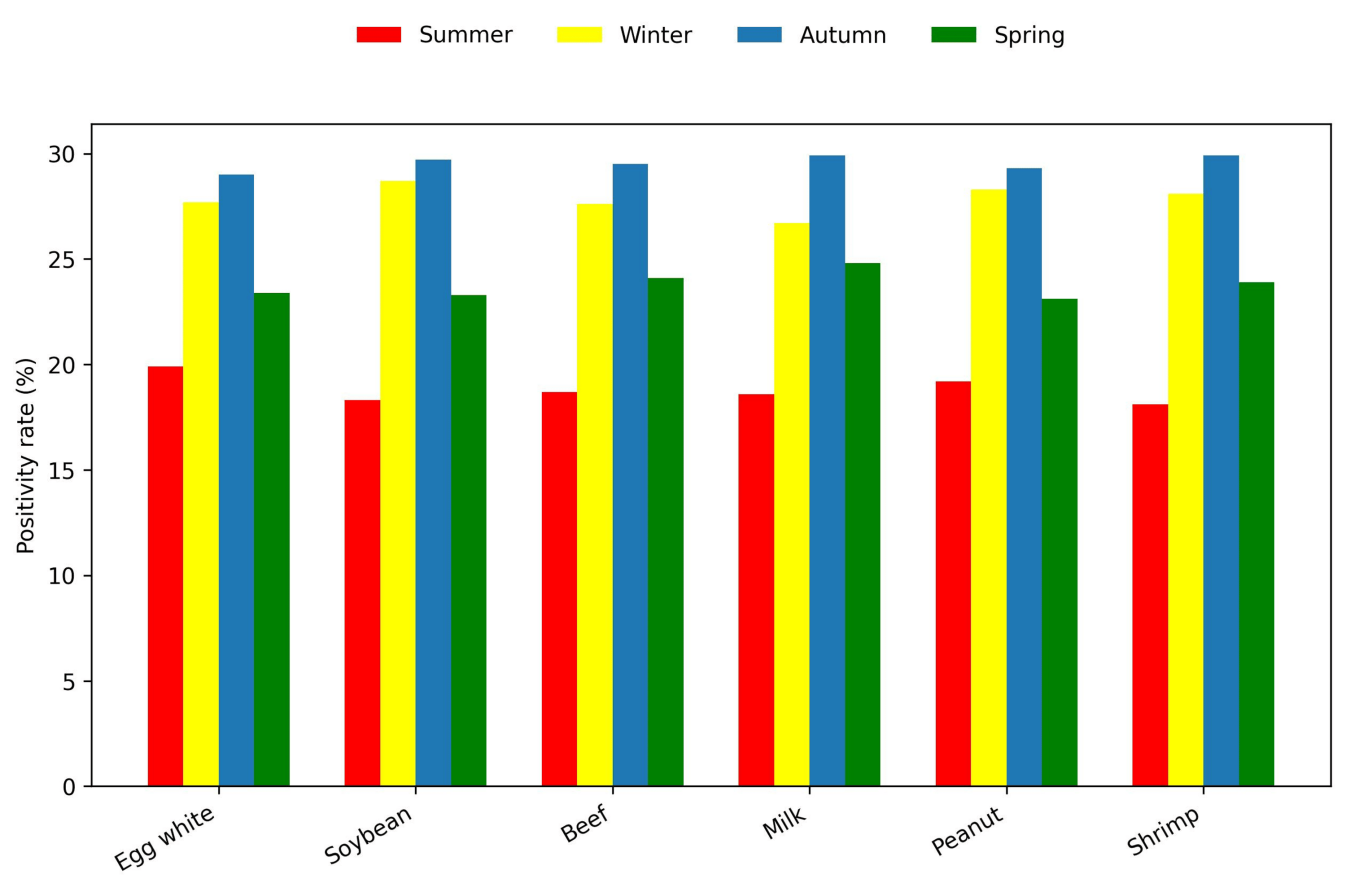
Food allergens by season

Overall, the most common food allergen was egg white (N = 841, 30%), followed by shrimp (N = 803, 28.7%) and peanut (N = 795, 28.4%) (Figure 1). The most common environmental allergen was *D. farinae *(N = 1,470, 52.5%), followed by *D. pteronyssinus *(N = 1,459, 52.1%) and cat epithelium (N = 841, 40.5%) (Figure 2). Environmental allergen positivity rates were highest in the <1-year age group (Figure 3), whereas food allergen positivity rates were highest in the 11-18-year age group (Figure 4). Both environmental and food allergens were most prevalent in autumn, followed by winter (Figure 5, Figure 6).

Both environmental and food allergen positivity rates were generally higher in males than in females. However, no gender-specific differences were observed for any food allergens or for *D. farinae *and *D. pteronyssinus*. Application of the Kruskal-Wallis test revealed statistically significant differences across all age groups, except for dog epithelium, soybean, and beef. The test also demonstrated statistically significant seasonal variation for *D. farinae*, *D. pteronyssinus*, *A. fumigatus*, and shrimp.

## Discussion

There is extensive evidence suggesting that food and environmental allergies are common and have been increasing globally over the past two to three decades. However, data from low- and middle-income countries remain limited, making it crucial to understand allergen dynamics in these settings, which differ not only in ethnicity but also in dietary patterns, environmental exposures, and climatic conditions. Our study provides a comprehensive laboratory-based analysis of food and environmental allergen sensitization patterns among children, including age- and season-related variations.

We found that the most common environmental allergen was *D. farinae*, followed by *D. pteronyssinus *and cat epithelium. A study conducted in South Korea reported similar findings, identifying *D. pteronyssinus *as the most common environmental allergen in children [14]. The predominance of house dust mites across diverse geographic settings suggests that indoor allergen exposure is a major contributor to pediatric sensitization. Climatic factors such as humidity and indoor crowding may further influence mite proliferation and allergen load.

Among food allergens in our study, the most common were egg white, followed by shrimp and peanut. A study from India reported similar results, showing increased sensitization to egg, prawn, and peanut among children, while also noting allergies to other foods such as brinjal, banana, and spinach in children aged 5-15 years [15]. Similarly, a review by Tedner et al. indicated that milk and egg were the most common food allergens in infants, while peanut and tree nuts predominated in older children [16]. Jeong et al. from Korea reported five major causative foods for immediate-type food allergies in children: cow’s milk, hen’s eggs, wheat, walnuts, and peanuts [17]. These differences may reflect variations in genetic susceptibility, dietary introduction practices, environmental exposures, and cultural dietary habits [18].

Our results demonstrated higher environmental allergen positivity rates in children under 1 year of age, while food allergen positivity was highest in the 11-18-year age group. Ying et al. reported that in children aged 1 month to 18 years, the most common food and environmental allergens were egg, milk, and house dust mites. They further noted that environmental allergens were more prevalent at older ages, whereas food allergies were more common in younger children [19]. Differences between studies may reflect variations in study design, referral patterns, environmental exposures, and the distinction between laboratory-detected sensitization and clinically confirmed allergy.

In our cohort, both environmental and food allergen positivity rates were generally higher in males than in females. Overall sensitization, including to mites, mixed grass pollens, and tree pollens, increased significantly with age. Male sex was strongly associated with sensitization, particularly in children under 8 years. After age 8, male predominance persisted, but a significant increase in sensitized females was also observed. Ying et al. similarly reported higher IgE-specific allergen positivity rates in males compared with females [19]. Biological explanations for sex differences may include hormonal influences and immune modulation, although the exact mechanisms remain incompletely understood.

The prevalence of environmental and food allergens was higher in autumn, followed by winter. Climate differences appear to influence the prevalence of specific environmental allergens. Similarly, Szekut et al. reported higher positivity rates for serum-specific allergens in children under 18 in Brazil, with most testing performed during spring (September to December) [20]. A multicenter study by Huang et al., conducted in preschool children, analyzed sensitization patterns to environmental allergens, including dust mites and multiple pollen types, and reported significant regional and seasonal differences. They found that *D. pteronyssinus *and *D. farinae *had the highest positivity rates, although peak levels were observed in May for Chinese preschool children, while pollen allergens were more prevalent in autumn [21]. These variations likely reflect differences in regional climate, humidity, indoor exposures, and environmental factors.

Most comparative data come from well-developed countries, highlighting a lack of robust data on food and environmental allergies in developing and low-middle-income countries. Potential explanations include limited access to diagnostic tools, high costs, and resource constraints. Epidemiological research is sparse, as public health priorities often focus on infectious diseases and malnutrition rather than chronic conditions like allergies. Socioeconomic barriers further delay recognition and management of allergic conditions, while urbanization and changing lifestyles are altering allergy patterns without corresponding data collection. These challenges contribute to significant gaps in understanding the prevalence and burden of allergies in these regions.

Importantly, this study is laboratory-based and includes only children who underwent allergen-specific IgE testing. Therefore, the findings reflect sensitization patterns within a tested cohort rather than population-level prevalence. Specific IgE positivity indicates immunologic sensitization and does not necessarily confirm clinical allergy, which requires appropriate clinical correlation.

## Conclusions

This study delineates the spectrum and distribution of food and environmental allergen sensitization among children undergoing allergen-specific IgE testing in Pakistan, with *Dermatophagoides* species and egg white identified as the predominant sensitizing allergens. Age-stratified analysis revealed higher environmental allergen positivity in younger children, whereas food allergen sensitization was more pronounced in adolescents. A distinct seasonal trend was observed, with increased sensitization rates during the autumn and winter months, suggesting potential environmental and exposure-related influences.

These findings characterize immunologic sensitization patterns within a clinically tested pediatric cohort and should not be extrapolated to estimate population-level prevalence. Importantly, the presence of allergen-specific IgE reflects sensitization rather than confirmed clinical allergy, which requires appropriate clinical correlation. Nevertheless, this large-scale laboratory-based analysis provides valuable regional data that may inform diagnostic strategies, enhance clinician awareness, and support context-specific approaches to allergy evaluation and management in similar resource-limited settings.
